# How Does Chinese Outward Foreign Direct Investment Respond to Host Country Cultural Tolerance and Trust?

**DOI:** 10.3389/fpsyg.2022.794455

**Published:** 2022-03-11

**Authors:** Haiyue Liu, Yuhan Wang, Qin Zhang, Jie Jiang

**Affiliations:** Business School, Sichuan University, Chengdu, China

**Keywords:** Chinese OFDI, cultural tolerance, cultural trust, rule of law, economic freedom index

## Abstract

Based on 2010 to 2019 Chinese outward foreign direct investment (OFDI) panel data from 39 host countries, this paper studies the relationships between host country cultural characteristics and Chinese OFDI. The OLS regression results show that the cultural tolerance and trust in the host countries are significantly positively correlated with Chinese OFDI, which are robust according to the system GMM tests. Further analysis reveals that cultural tolerance is more positively related to Chinese OFDI in host countries with higher legislation and economic freedom, while cultural trust is positively associated with Chinese OFDI in host countries with lower legislation and economic freedom. In addition, higher cultural tolerance and trust promote Chinese OFDI in countries with greater cultural distance. Unlike traditional studies based on cultural distance in international trade, using more representative cultural characteristics, this paper provides references to Chinese OFDI decision-making based on the root characteristics associated with heterogeneous cultural influences.

## Introduction

With the deepening of economic globalization, foreign direct investment (FDI) has become essential for economic interactions among countries. Consequently, the Chinese government has actively encouraged sustained and rapid outward foreign direct investment (OFDI). According to the report issued by the Chinese Ministry of Commerce, Chinese OFDI flow was ranked first in the world in 2020, and its stock was ranked third; therefore, the Chinese OFDI has become globally influential.

With the encouragement of “Going Global Strategy”^1^ and “the Belt and Road Initiative,” a growing number of Chinese enterprises have actively implemented OFDI. By the end of 2020, more than 28,000 Chinese domestic investors will have established 45,000 OFDI enterprises in 189 countries (regions). Since then, Chinese FDI has expanded in scale and scope.

The characteristics of many host countries that attract Chinese FDI vary greatly in social development levels, richness, diversity, and especially in national cultures. The host country of Chinese foreign investment almost covers the eight civilizations described by Samuel Huntington (Chinese, Japanese, Indian, Islamic, Western, Orthodox, Latin American, and African societies). The integration and convergence of cultures can bring new opportunities for trade development. However, cultural differences can also result in cultural clashes and affect healthy trade relationships.

Therefore, the effects of these cultural differences on investment need to be defined to furtherly optimize Chinese OFDI. Due to its uncertainties, culture has always been regarded as an exogenous factor. Since the 1980s, cultural distance has been used to describe the cultural differences between two countries. However, the cultural distance between the two countries has been fixed for many years. If we only consider the effect of cultural factors from the perspective of cultural distance, the understanding of the cultural environment of the host country when investing abroad will be limited. If other critical cultural factors are ignored when seeking OFDI opportunities, China may find it challenging to respond dynamically and make accurate foreign investment decisions.

There are two apparent limitations in previous research on the relationship between cultural distance and Chinese OFDI. Firstly, there is no consensus on the relationship between the two. Some research has concluded that cultural distance has hindered Chinese OFDI (Ding and Li, 2017; Han and Jiang, 2017; Wang and Wang, 2019; Li et al., 2020). While some studies have stated that this relationship is U-shaped, Liu et al. (2018) point out that the growth rates for “liability of foreignness” (LOF) and “advantages of foreignness” (AOF) ultimately determine whether cultural distance will lead to conflicts or promote innovation. However, other research has even found that there are no significant correlations (Ren and Yang, 2020). Secondly, the studies find that on the different cultural dimensions, bilateral cultural distance has had different degrees of inhibition on OFDI (Liu et al., 2018; Lin and Liu, 2020). Shenkar insists that conceptual and methodological deficiencies exist in studying FDI involving cultural distance. The cultural distance concept has embraced an equivalence assumption that regards all cultural dimensions as equal and has ignored the significant conceptual impacts of specific cultural dimensions (Shenkar, 2001).

Since the effect of cultural distance has generally been examined based on samples from the home and host countries, it is hard to judge whether cultural distance drives the results (Brouthers et al., 2016). Taking the influence of cultural distance on the trade entry mode choice to test the relevance of the cultural distance concept, some studies have found that the explanatory power of cultural distance is minimal, which extends this argument to the entire field of international business (Harzing and Pudelko, 2016). Previous research also overestimates cultural distance’s role and inappropriately attributes causality to cultural distance. It has also been found that after the introduction of cultural characteristics, most distance effects are no longer significant as the cultural characteristics provide more reasonable explanatory factors. Therefore, more in-depth cultural characteristics studies are needed to avoid the illusion of causality (Kirkman et al., 2006; Tung and Verbeke, 2010). At the same time, Satir’s iceberg theory proposes that if culture is equated with an iceberg, the visible part above the water is the surface or logo culture, such as creative cultural products and artistic works, and the part under the water, which cannot be directly observed, is the foundation or the deep cultural characteristics, such as the values and ways of thinking. It is the source of heterogeneous cultural influences. As an intrinsic and relatively stable part of the culture, cultural characteristics play critical roles in an organization’s development; therefore, the characteristics of culture itself may be more influential than the distance.

As a formal, impartial, and equitable global survey, The World Value Survey (WVS) publishes data and reports that have been used in more than 1,000 papers in 20 languages. Therefore, to examine the LOF that multinational enterprises may encounter in foreign markets and investigate the effects on the establishment of friendly cooperative international exchanges, WVS data is used in this paper to assess the influence of cultural characteristics on Chinese OFDI. Cultural trust is the foundation of successful cooperation. Sustained and stable collaboration is based on cultural trust, and only mutual trust can genuinely help establish cooperative relations. Since the cooperation parties will inevitably chase different goals, frictions, and contradictions will always occur in collaboration, and cultural tolerance is also needed to develop cooperation based on cultural trust. Tolerance is closely related to trade and investment in economic globalization and connected to cooperation (Berggren and Nilsson, 2015). It is assumed that tolerant cultures are more likely to promote high-level cooperative relationships between two countries, causing less conflict, and partners are more willing to form deeper collaborative relationships and have a higher degree of trust (Eriksson et al., 2021). Cooperation, tolerance, and trust are essential for practical OFDI requirements. Therefore, in this study, host country cultural tolerance and trust are selected as the test dimensions to assess the OFDI specificity, and we explore the moderating roles of the rule of law, economic freedom, and cultural distance in the above relationships. It is found that host country cultural tolerance and trust are significantly positively related to Chinese OFDI. In further analysis, it is also found that cultural tolerance is more positively related to Chinese OFDI in host countries with a higher rule of law and economic freedom, while cultural trust is positively associated with Chinese OFDI in host countries with a lower rule of law and economic freedom. Higher cultural tolerance and trust promote Chinese OFDI in countries with greater cultural distance.

This study makes three main contributions. Firstly, we find conflicting conclusions in prior studies on the relationship between cultural distance and Chinese OFDI. The host country’s cultural characteristics are more likely to be better explanatory factors by optimizing the concept and measurement of cultural characteristics, which provides a valuable supplement to Chinese OFDI research from the perspective of culture. Secondly, unlike the cultural distance notions propounded by Hofstede et al., this paper focuses on the relationship between cultural tolerance and trust in host countries and Chinese OFDI. It identifies the specific host country cultural characteristics of cultural tolerance and trust as an essential formerly ignored driver of investment decision-making. The moderating role of the rule of law and economic freedom are also found to be influential and divergent related to cultural tolerance and trust. Thirdly, the current cultural distance framework and measurement method are extended by using the more representative cultural characteristics to analyze its effect on Chinese OFDI, which may also have good explanatory power for other international trade phenomena.

## Theoretical Analysis and Hypothesis

### Host Country Cultural Tolerance and Chinese OFDI

Tolerance responds to free expression and peaceful coexistence when faced with conflicting cultures, beliefs, and lifestyles. Because of the diversity and complexity of cultures, there have been many discussions on multiculturalism and social pluralism. Tolerance is regarded as a kind of restraint that the subject has the ability to intervene but does not actually take action against the hostile others to ensure peaceful coexistence (Cohen, 2004; Liu and Zhao, 2007).

When conducting OFDI, transnational enterprises are often faced with foreignness due to information asymmetry and legitimacy insufficiency. This outsider identity can often hinder multinational enterprises from collecting information and acquiring resources, making the OFDI decisions difficult (Cai, 2020). However, higher cultural tolerance of host countries can increase social diversification and reduce communication and information costs (Lu and Zhao, 2019). In more tolerant cultural environments, foreignness restrictions for transnational enterprises have been reduced. The cultural tolerances in host countries can affect the degree of contradictions and conflicts caused by cultural differences between the two countries and can benefit or adversely affect foreign investment decisions.

Institutions are regarded as a series of “rules of the game” including formal and informal institutions and are involved in various aspects of a country’s political, legal, economic, and social system (North, 1990). Institutional Theory proposes that the institutional environment is composed of a country’s political systems, economic systems, social rules, value systems, and education systems that will significantly affect enterprises’ business activities. When multinational enterprises enter the host country, they must first adapt to the institutional environment of the host country to obtain legitimacy. The legitimacy in Institutional Theory emphasizes the importance of acceptance and tolerance in local societies and groups. However, cultural differences in informal rules are not conducive to the legitimacy in host countries (Kostova and Zaheer, 1999; Lu and Xu, 2006). Generally, countries with broad tolerances are more willing to accept people from different backgrounds into their social and economic life and are more likely to provide open, tolerant cultural environments for foreign investors (Florida, 2010; Ang et al., 2015). It is also beneficial for transnational corporations to be recognized by organizations or groups in their target international outward investment country.

Inclusive political systems presuppose political pluralism. Politically pluralist societies are tolerant, equal, and open to all, and these governments also promote cultural tolerance and the establishment of inclusive, open social environments (He, 2014). Due to their focus on social-cultural development, politically pluralist societies often implement fair trading rules and public services to develop the foreign investment market.

Therefore, the stronger a country’s cultural tolerance, the more inclusive the political environment for foreign investment. In the host countries with higher cultural tolerance, local stakeholders are more willing to recognize the heterogeneous identity of foreign enterprises and less likely to label transnational investors as foreign and deny their institutional legitimacy. Given this discussion, this paper proposes the following hypothesis:

*Hypothesis 1:* The higher the host country’s cultural tolerance, the greater the Chinese OFDI.

### Host Country Cultural Trust and Chinese OFDI

Trust is defined by Fukuyama as an expectation of normal honest, cooperative behavior. Trust plays a vital role in developing secure, stable social partnerships (Lange and Am, 2015), and trust in cultural traits can ensure good cooperative relationships (Chen et al., 2021), bring communication benefits, and reduce information asymmetry. Therefore, trust positively impacts foreign investment activities (Chi and Wang, 2020; Zhu et al., 2020).

People in high-trust societies tend to perceive others as trustworthy and think it is unnecessary to control or check others’ behavior. Because individuals generally develop relationships between organizations and within the organization (Aulakh et al., 1996), interpersonal trust (between individuals) is regarded as the basis of trust between organizations (Dyer and Chu, 2000). The positive effects of trust can also extend to the inter-organizational and social levels as trusted interpersonal connections can assist in developing further safe social relationships. Trust promotes cooperation, development, and cohesion between members, reduces conflicts, and improves efficiency (Navarro-Carrillo et al., 2018). Therefore, high levels of interpersonal trust impact both individual trust and inter-organizational trust in the host countries.

We think that the degree of the host country’s trust can affect cooperative tendencies and enhance the confidence for Chinese enterprises to conduct OFDI. Moreover, the two reasons for its promotion effect are as follows: (1) High interpersonal trust engenders sociability, bringing new cooperative relationships. These social connections then increase the speed of forming new networks (Porta et al., 1997), facilitating friendly investment relations between China and the host countries. (2) Since behavior in high-trust societies is based on cooperation rather than opportunism, trust does not inhibit interpersonal relationships (Hill, 1990).

Therefore, when there are high levels of host country cultural, personal, and inter-organizational trust, the information asymmetry costs are lower and it is easier for foreign investors to develop social and business relationships, all of which will make the host country more attractive to FDI. Thus, the following hypothesis is proposed:

*Hypothesis 2:* The higher the host country’s cultural trust, the greater the Chinese OFDI.

## Empirical Study

### Data

The data on the host country’s cultural traits were obtained from the World Values Survey, which covers a small number of countries and regions. To ensure the availability and completeness of the data on other variables, this paper refers to Chinese OFDI data in 39 host countries published in the Statistical Bulletin of Chinese OFDI from 2010 to 2019. Tax havens, such as the British Virgin Islands, the Cayman Islands, and Bermuda, were excluded, as sampled from Hong Kong, Macao, Taiwan, and regions where critical data were missing (Zuo and Yang, 2021).

The host country’s cultural tolerance (*Evi*) and trust (*Tru*) data were obtained from WVS. The data from 2010 to 2014 were collected from WVS 6 (Inglehart et al., 2014), the data from 2015 and 2016 were collected from WVS 6 and WVS 7 averages,^2^ and the data from 2017 to 2019 were collected from WVS 7 (Haerpfer et al., 2020). The cultural distance (*Dcult*) data in 2015 were obtained from Geert Hofstede’s website. The geographical distance was derived from the CEPII database in France and represented as the distance between two capitals multiplied by the international oil price obtained from the IMF database. The difference between the logarithms of GDP of the two countries (*GDP2*), FDI openness (*Infdi*), financial development (*Fd*), manufacturing level (*Manu*), transport service (*Trans*), economic development (*Ed*), infrastructure (*Inter*), mineral and metal resources (*Mr*), fuel resources (*Fr*) were extracted from the World Bank (WDI) database. To weaken the influence of the extreme data values, all continuous variables were treated before and after with a 1% tail reduction.

### Variable Descriptions

The explained variable *LnOFDI* represents the scale of Chinese OFDI. Two accounting methods are used to measure OFDI: flow and stock. Using stock data for quantitative analysis can avoid any multicollinearity between trade and investment and reflect the investment lag effect. Therefore, OFDI stock data is adopted in this paper. Specifically, the logarithms of the OFDI stock data reported in the Chinese annual outbound investment bulletins are taken to reduce the impact of heteroscedasticity.

The explanatory variable *Evi* represents the host country’s cultural tolerance. Referring to research of Lu and Zhao (2019) and Zuo and Yang (2021), we use the Emancipative Values Index from WVS to represent the attitudes toward minority groups or social phenomena. Based on various dimensions of equality, openness, or exclusion attitudes, this Index indicates the cultural tolerance in the host countries, and the higher the equal and open attitude proportion, the higher the host country’s cultural tolerance (Lu and Zhao, 2019; Zuo and Yang, 2021).

The explanatory variable *Tru* represents the host country’s cultural trust. According to the study of Zhao and Chi (2014), the WVS cultural trust index is used in our paper to assess the cultural trust in the host countries, and the more significant the proportion of people who believe that they could trust people, the higher the degree of cultural trust in the host countries (Zhao and Chi, 2014).

The control variables include cultural distance (*Dcult*), geographic distance (*Dgeo*), *GDP2*, FDI openness (*Infdi*), financial development (*Fd*), manufacturing level (*Manu*), transport service (*Trans*), economic development (*Ed*), infrastructure (*Inter*), mineral and metal resources (*Mr*), and fuel resources (*Fr*).

Cultural distance is included in the control variables to exclude the possible impacts of its dimensions on Chinese OFDI. Referring to Hofstede’s national culture concept and study of Kogut and Singh (1988), cultural distance is measured by the cultural distance index proposed by Kogut and Singh (1988). Then, the six dimensions’ cultural indicators are synthesized into comprehensive indicators to reflect the cultural distance, and the distance is measured by the Kolmogorov–Smirnov index (KSI; Chen, 2020). The distance between the two countries’ capital cities is multiplied by the international oil price to indicate the geographical distance (Jiang and Jiang, 2012), and the difference between the GDP logarithms is used to represent the GDP differences between China and the host country. The proportion of annual net FDI inflow in the host country’s GDP is used as the proxy variable of FDI openness, and the proportion of private domestic credit by the financial sector in the host country’s GDP is used to represent the financial development (Munemo, 2017). The manufacturing level is the proportion of the manufacturing added value in the host country’s GDP. The transport service is the proportion of transport service of the host country in commercial service export, and the economic development is measured by annual per capital GDP growth rate. We use internet access per 100 people in the host countries to evaluate the level of infrastructure construction (Yuan et al., 2018), and the natural resources are used as the percentage share of the host country’s mineral, metal, and fuel exports in the overall commodity exports.

### Research Design

Gorter and Poyhonen’s gravity model has been widely applied to explain cross-border trade and capital flows (Markusen and Maskus, 2002; Kahouli and Maktouf, 2015). Therefore, the cultural characteristics and control variables are introduced based on the above model.

To test the relationship between the host country’s cultural tolerance and Chinese OFDI stock, the main regression model is established as follows:


$$\begin{array}{l} \mathrm{LnOFD}I_{jt}=\alpha_{0}+\alpha_{1}Evi_{jt}+\alpha_{2}\mathrm{Dcul}t_{jt}+\alpha_{3}Dgeo_{jt}\\ \;+\alpha_{4}GDP2_{jt}+\alpha_{5}\mathrm{Infd}i_{jt}+\alpha_{6}{Fd}_{jt}+\alpha_{7}\mathrm{Man}u_{jt}\\ \;+\alpha_{8}\mathrm{Tran}s_{jt}+\alpha_{9}{Ed}_{jt}\;+\alpha_{10}\mathrm{Inte}r_{jt}+\alpha_{11}{Mr}_{jt}\\ \;+\alpha_{12}{Fr}_{jt}+\sum Year_{t}+\sum \mathrm{Contien}t_{j}+\varepsilon_{jt} \end{array}$$

To test the relationship between the host country’s cultural trust and Chinese OFDI stock, the main regression model is established as follows:


$$\begin{array}{l} \mathrm{LnOFD}I_{jt}=\beta_{0}+\beta_{1}Tru_{jt}+\beta_{2}\mathrm{Dcul}t_{jt}+\beta_{3}Dgeo_{jt}\\ \;+\beta_{4}GDP2_{jt}+\beta_{5}\mathrm{Infd}i_{jt}+\beta_{6}{Fd}_{jt}+\beta_{7}\mathrm{Man}u_{jt}\\ \;+\beta_{8}\mathrm{Tran}s_{jt}+\beta_{9}{Ed}_{jt}+\beta_{10}\mathrm{Inte}r_{jt}+\beta_{11}{Mr}_{jt}\\ \;+\beta_{12}{Fr}_{jt}+\sum Year_{t}+\sum \mathrm{Contien}t_{j}+\varepsilon_{jt} \end{array}$$

where the subscripts *j* and *t*, respectively, represent the host country *j* and year *t*. Coefficients *α_1_* and *β_1_* test the influence of cultural tolerance or trust of the host country on the Chinese OFDI stock, and *ε_jt_* is a stochastic error term. *Year_t_* is introduced into the model to control the potential impact of global annual macroeconomic changes on Chinese OFDI and the *Continent_j_* controls the influence of unobservable heterogeneity of the host country to indicate Asia, Africa, Europe, North America, South America, and Oceania (Huang and Lin, 2020).

## Empirical Results

### Descriptive Statistics

The descriptive statistics for the explained variables, explanatory variables, and control variables are shown in Table 1, indicating that the average Chinese OFDI stock value for the explained variables (*LnOFDI*) is 10.888, and the maximum is 15.723, while the minimum is 4.883. It is also shown that there are significant differences in the Chinese OFDI in the various host countries. The mean cultural tolerance (*Evi*) in the host countries is 0.434, and the maximum is 0.740, while the minimum is 0.240. The mean cultural trust (*Tru*) in the host countries is 0.236, and the maximum is 0.721, while the minimum is 0.041.

**Table 1:** Descriptive variable statistics.

Variable	Obs	Mean	Std. Dev.	Median	Min	Max
LnOFDI	284	10.888	2.152	11.078	4.883	15.723
Evi	284	0.434	0.118	0.428	0.240	0.740
Tru	284	0.236	0.155	0.222	0.041	0.721
Dcult	284	3.784	1.840	3.469	0.697	7.762
Dgeo	284	13.105	0.700	13.183	10.790	14.420
GDP2	284	3.597	1.839	3.536	−0.641	7.017
Infdi	284	6.332	20.688	2.558	−8.487	146.727
Fd	284	75.751	55.594	55.167	5.908	249.918
Manu	284	2.744	4.670	2.301	−11.923	19.321
Trans	284	25.354	12.611	24.007	5.908	61.547
Ed	284	2.178	2.766	2.025	−6.794	9.672
Inter	284	58.966	25.034	62.306	7.000	96.358
Mr	284	9.586	14.263	3.753	0.000	59.315
Fr	284	15.094	22.668	6.201	0.000	99.797

### Correlation Analysis

The correlation coefficient matrix of variables is shown in Table 2. The collinearity between the variables is not severe, with the preliminary results showing that the cultural tolerance and trust degrees in the host countries are significantly and positively correlated with Chinese OFDI stock.

**Table 2:** Correlation coefficient matrix of variables.

	LnOFDI	Evi	Tru	Dcult	Dgeo	GDP2	Infdi	Fd	Manu	Trans	Ed	Inter	Mr	Fr
LnOFDI	1													
Evi	0.331^***^	1												
Tru	0.257^***^	0.662^***^	1											
Dcult	−0.378^***^	−0.262^***^	−0.142^**^	1										
Dgeo	−0.159^***^	0.043	−0.330^***^	0.239^***^	1									
GDP2	−0.735^***^	−0.395^***^	−0.209^***^	0.571^***^	0.046	1								
Infdi	−0.189^***^	−0.019	−0.223^***^	0.388^***^	0.056	0.268^***^	1							
Fd	0.302^***^	0.523^***^	0.378^***^	−0.010	−0.279^***^	−0.290^***^	0.441^***^	1						
Manu	−0.124^**^	−0.181^***^	−0.080	0.057	0.081	0.119^**^	−0.216^***^	−0.267^***^	1					
Trans	−0.196^***^	−0.202^***^	−0.061	0.088	−0.066	0.189^***^	0.091	−0.119^**^	−0.019	1				
Ed	−0.131^**^	−0.084	−0.101^*^	−0.090	0.054	0.068	−0.151^**^	−0.243^***^	0.739^***^	0.087	1			
Inter	0.213^***^	0.731^***^	0.500^***^	−0.175^***^	−0.280^***^	−0.204^***^	0.035	0.546^***^	−0.252^***^	−0.081	−0.174^***^	1		
Mr	−0.285^***^	−0.118^**^	−0.336^***^	0.112^*^	0.363^***^	0.314^***^	0.064	−0.060	0.030	0.083	0.111^*^	−0.162^***^	1	
Fr	0.029	−0.189^***^	0.001	0.156^***^	0.067	−0.029	−0.077	−0.276^***^	0.142^**^	0.055	0.051	−0.146^**^	−0.241^***^	1

*** *p* < 0.01.

** *p* < 0.05.

* *p* < 0.1.

**Figure 1:**
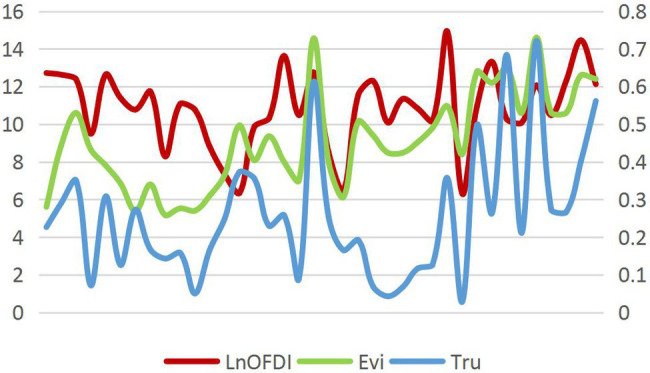
Relationship between host country’s cultural tolerance, cultural trust, and Chinese outward foreign direct investment (OFDI) stock.

The means for the host country’s cultural tolerance, cultural trust, and the Chinese OFDI stock are put into a line chart to explore the relationships. As shown in Figure 1, there is a possible positive correlation between the host countries’ cultural tolerance, cultural trust, and the Chinese OFDI stock. Therefore, an empirical model is built to further explore these relationships.

### Main Regression Results

Table 3 gives the test results for hypothesis H_1_ and hypothesis H_2_. The host country’s cultural tolerance (*Evi*) is added into model 2, and its coefficient is 5.528, which is significant at the 1% level. After controlling other variables, the host country’s cultural tolerance is positively correlated with Chinese OFDI stock; the higher the host country’s cultural tolerance, the greater the Chinese OFDI stock. Hypothesis H_1_ is approved. The host country’s cultural trust (*Tru*) is added into model 3, and its coefficient is 2.765, significant at the 1% level. There is a significantly positive relationship between the host country’s cultural trust and Chinese OFDI stock, and the higher the host country’s cultural trust, the larger the Chinese OFDI stock. Hypothesis H_2_ is confirmed.

**Table 3:** Main regression results.

LnOFDI	(1) Basic model	(2) Tolerance model	(3) Trust model
Evi		5.528^***^	
		(3.75)	
Tru			2.765^***^
			(3.12)
Dcult	0.106	0.092	0.087
	(1.15)	(1.09)	(0.96)
Dgeo	−0.323	−0.368	−0.226
	(−1.39)	(−1.59)	(−0.98)
GDP2	−1.020^***^	−0.983^***^	−1.023^***^
	(−11.74)	(−11.36)	(−11.47)
Infdi	0.001	0.004	0.009
	(0.19)	(0.73)	(1.42)
Fd	0.006^***^	0.002	0.003
	(2.63)	(0.70)	(0.98)
Manu	0.044	0.051^*^	0.047
	(1.44)	(1.78)	(1.56)
Trans	0.004	0.013^*^	0.006
	(0.53)	(1.86)	(0.98)
Ed	−0.041	−0.063	−0.035
	(−0.90)	(−1.43)	(−0.77)
Inter	−0.025^***^	−0.035^***^	−0.026^***^
	(−4.06)	(−5.78)	(−4.59)
Mr	−0.000	0.002	0.001
	(−0.02)	(0.30)	(0.09)
Fr	0.002	0.004	−0.000
	(0.39)	(0.90)	(−0.08)
Year	Yes	Yes	Yes
Continent	Yes	Yes	Yes
Constant	17.130^***^	16.053^***^	15.570^***^
	(5.66)	(5.22)	(5.15)
*N*	284	284	284
*R*^2^_a	0.692	0.708	0.704
*F*	27.25	29.21	29.39

*** *p* < 0.01.

* *p* < 0.1, the *t* statistic is in parentheses.

### Robustness Test

#### Change the Variable Interpretation Method

The cultural tolerance and trust measurement methods are altered to obtain more robust empirical results. Florida (2014) finds that homosexuality acceptance is a leading indicator of a place’s tolerance (Florida, 2014). Since they can accept homosexuals, they have a more tolerant attitude toward other groups. We use an alternative measurement of homosexuality acceptance (HomoACPT) from the World Values Survey to measure cultural tolerance. From the original WVS cultural trust measurement method based on the question “Do you trust most people?,” the mean answer value for the following six questions is used to measure the level of in-group trust and the level of out-group trust (Delhey et al., 2011), which are denoted as Tru6: (a) Do you trust your family?; (b) Do you trust your neighbors?; (c) Do you trust people who know you well?; (d) Do you trust people you meet for the first time?; (e) Do you trust people of other religions?; and (f) Do you trust people from different nationalities?

Table 4 shows the results of using different measurements of explanatory variables, and the results are consistent with previous conclusions, which demonstrate the robustness of the basic empirical results.

**Table 4:** Change variable interpretation results.

Change the interpretations of cultural tolerance and trust		
HomoACPT	2.189^***^	
	(3.37)	
Tru6		3.864^***^
		(2.87)
Controls	Yes	Yes
*N*	284	281
*R*^2^_a	0.704	0.698
*F*	26.47	27.51

*** *p* < 0.01. the *t* statistic is in parentheses.

#### GMM Two-Stage Regression

Due to the two-way causality and omitted variables in the empirical model and the possibility that the host country’s cultural tolerance, trust, and other factors may have long-term effects on Chinese OFDI stock, potential endogenous problems should be considered. Because of possible self-selection bias concerns, we employ the dynamic panel system GMM two-stage approach to test the robustness of the cultural trust in OFDI. The explanatory variables in the earlier stage and the explained variables in the later stage are used as instrumental variables to minimize the endogeneity influences on the parameter estimations.

The results are shown in Table 5. The Sargan overidentification test and the AR (2) residual non-autocorrelation test are all passed, showing the effectiveness of the system GMM method. The host country’s cultural tolerance coefficient is significantly positive at the 5% level, and the host country’s cultural trust coefficient is still significantly positive at the 1% level. The results further document that our key findings on the beneficial impact of cultural tolerance and trust on OFDI stock are robust and reliable.

**Table 5:** Two-stage regression results for the system GMM.

System GMM		
L.LnOFDI	0.890^***^	0.891^***^
	(43.74)	(36.03)
Evi	1.534^**^	
	(2.05)	
Tru		0.948^***^
		(3.19)
Controls	Yes	Yes
*N*	246	246
Sargan test-*p*-value	0.516	0.304
Hansen test-*p*-value	0.875	0.998
AR (1)	0.004	0.006
AR (2)	0.508	0.375

*** *p* < 0.01.

** *p* < 0.05. the *t* statistic is in parentheses.

## Further Analysis

Transnational enterprises need to abide by the host country’s rules to achieve healthy and stable development. Institutional rules include formal ones such as laws and regulations and informal ones such as cultural customs (Casson et al., 2010). The new theory of institutional economics believes that formal institutions play a role only when they are recognized by informal institutions, which is an essential factor in the operation of formal institutions (Andriani and Bruno, 2021). Good institutional rules establish a standardized market environment and promote orderly investment trading activities (Alam et al., 2019).

The formal institution mainly includes political institutions and economic institutions (Estrin et al., 2009). The legal institution is the core part of the political institution. A complete, adequate, and transparent legal system in the host country can protect the income of assets and the rights and interests of individuals, thereby encouraging direct investment. Countries with better economic systems often enjoy a less complex economic environment, so it is easier for countries to take adequate measures to reduce the transaction costs of enterprises significantly. In this paper, the rule of law level is taken as a representative factor of the political institution. The degree of economic freedom is the main economic institution factor. The moderating effects of these two factors on the host country’s cultural characteristics and Chinese OFDI are therefore analyzed to assess the different influences of host country cultural tolerance and trust on Chinese OFDI under different formal institutional environments. In the new institutional economics, cultural factors are an essential part of informal institutions. Existing studies usually use cultural distance as the proxy variable of informal institutional distance. Higher cultural distance increases the uncertainty and costs of OFDI and threatens the survival and performance of international joint ventures, which could restrain Chinese OFDI (Ding and Li, 2017; Han and Jiang, 2017; Wang and Wang, 2019; Li et al., 2020). This paper will also further research whether tolerance and trust of the host country’s cultural characteristics can alleviate “liability of foreignness” (LOF) caused by cultural distance and become a factor to attract investors.

### Influence of Host Country Cultural Characteristics on Chinese OFDI Under Different Rule of Law Levels

Countries with different rule of law levels have different market norms and appropriate transaction transparency levels. Complete legal systems and solid legal binding forces can reduce opportunistic behavior and investment risks and protect the asset income and rights of the investing country (Bailey, 2017; Dupont et al., 2020). As host country cultural tolerance and cultural trust may affect Chinese OFDI, the Rule of Law Index of the World Governance Indicators (WGI) is selected to assess the rule of law levels in the host countries, and the higher the index, the higher the rule of law level. The sample countries are then divided into two groups (*Law* = 1 and *Law* = 0) based on each country’s median rule of law levels.

Table 6 shows the effect of host country cultural characteristics on Chinese OFDI stock at the different rule of law levels. It can be seen that in countries with a higher rule of law level, the host country’s cultural tolerance and Chinese OFDI coefficient is 8.207 and is significantly positive at the 1% level. However, the host country’s cultural trust does not affect Chinese OFDI substantially. In countries with a lower rule of law level, the host country’s cultural tolerance has no significant impact on Chinese OFDI. Nevertheless, the host country’s cultural trust and Chinese OFDI coefficient is 6.607, which is significant at the 1% level.

**Table 6:** Grouping regression results based on differences of rule of law level and economic freedom in host countries.

	(1)	(2)	(3)	(4)	(5)	(6)	(7)	(8)
	Law = 1	Law = 0	Law = 1	Law = 0	EFI = 1	EFI = 0	EFI = 1	EFI = 0
Evi	8.207^***^	5.568			7.544^***^	−4.868		
	(3.71)	(0.91)			(2.90)	(−1.38)		
Tru			0.691	6.607^***^			−0.357	7.206^***^
			(0.51)	(3.76)			(−0.24)	(5.29)
Controls	Yes	Yes	Yes	Yes	Yes	Yes	Yes	Yes
*N*	143	141	143	141	138	139	138	139
*R*^2^_a	0.819	0.744	0.791	0.778	0.796	0.753	0.779	0.792
*F*	73.84	23.08	61.00	31.31	34.16	33.41	33.93	39.99

*** *p* < 0.01. the *t* statistic is in parentheses.

Chinese enterprises may not adapt to the strict legislation environments when the host country is under a higher rule of law level. In this case, Chinese investors are more likely to invest in a host country with a higher degree of cultural tolerance, thus helping Chinese enterprises offset the efficiency loss and integration difficulties brought by the higher level of the rule of law in the host country. Highly trusting societies rely less on the formal system when implementing agreements, and trust between people can function as formal systems to a certain extent. When facing a lower level of the rule of law in a host country, that is, the legislation is not complete or not transparent, and the information asymmetry is intensified, Chinese enterprises would spend more resources to find information and learn local hidden rules, thus increasing operational uncertainty. Under this condition, cultural trust plays an essential role in developing Chinese OFDI. In host countries with a lower rule of law, cultural trust plays a more prominent role in promoting Chinese OFDI.

### Influence of Host Country Cultural Characteristics on Chinese OFDI Under Different Degrees of Economic Freedom

As economic freedom can affect the host country market environment, it can affect Chinese OFDI decisions (Economou, 2019; Lu et al., 2020), that is, as the market environment tolerances of countries with different degrees of economic freedom vary, the host country’s cultural characteristics may also affect Chinese OFDI. The Economic Freedom Index (*EFI*) published by the Heritage Foundation is used to assess the host country’s economic environments. The Index has a 100-point scale, with higher scores indicating great economic freedom. Taking the median Economic Freedom Index in each country as the boundary, the sample countries are divided into higher economic freedom (*EFI* = 1) and lower economic freedom (*EFI* = 0).

The results in Table 6 show that in host countries with higher economic freedom, the cultural tolerance coefficient is 7.544, which is significant at the 1% level. However, the cultural trust degree of the host country has no significant influence on Chinese OFDI. In host countries with lower economic freedom, the cultural trust coefficient is 7.206 and is significantly positive at the 1% level. Nevertheless, the host country’s cultural tolerance shows no significant impact on Chinese OFDI stock.

A country with higher economic freedom will attract foreign investors more. Nevertheless, resources are always limited. There are blindness and limitations for investors in allocating resources, triggering vicious competition among investors. Therefore, the tolerance characteristic in the host country’s culture could guide foreign enterprises to uphold the concept of friendly coexistence and avoid unfair and moral behaviors when competition and conflict occur, resulting in more foreign direct investment. On the contrary, more government intervention might distort the market signal in a host country with lower economic freedom. Faced with a low level of the host country’s cultural trust, investors’ signal acquisition and decision-making will be even more difficult. A higher degree of trust can partially alleviate the dilemma caused by insufficient economic freedom, thereby attracting more investment from Chinese enterprises.

### Influence of Host Country Cultural Characteristics on Chinese OFDI Under Different Cultural Distance

In countries with higher cultural distance, multinational enterprises will encounter more substantial barriers to the flow of information and production factors, thus inhibiting the development of the investment. Tolerance and trust in the cultural characteristics of the host country will act as means of mitigating the obstacles to OFDI. Using the median cultural distance of all countries as the boundary, the sample countries are divided into the higher cultural distance (*Dcult* = 1) group and lower cultural distance (*Dcult* = 0) group for comparative analysis.

As seen in Table 7, in host countries with higher cultural distance, the cultural tolerance coefficient is 10.569, and the cultural trust coefficient is 7.231, both of which are significantly positive at the 1% level. However, in host countries with lower cultural distance, the host country’s cultural tolerance and trust have no significant impact on the Chinese OFDI. It shows that cultural tolerance and trust are positively related to Chinese OFDI in host countries with higher cultural distance, and the marginal effect of cultural tolerance and trust is more pronounced in countries with higher cultural distance than those with lower cultural distance.

**Table 7:** Grouping regression results based on cultural distance differences in host countries.

	(1)	(2)	(3)	(4)
	Dcult = 1	Dcult = 0	Dcult = 1	Dcult = 0
Evi	10.569^***^	0.400		
	(4.68)	(0.24)		
Tru			7.231^***^	0.973
			(3.80)	(1.27)
Controls	Yes	Yes	Yes	Yes
*N*	141	143	141	143
*R*^2^_a	0.753	0.798	0.736	0.800
*F*	35.73	30.83	42.58	31.15

*** *p* < 0.01, the *t* statistic is in parentheses.

It can be explained that when the cultural distance between the two countries is relatively higher, it will be more challenging for Chinese enterprises to adapt to local culture. Under these circumstances, the tolerance and trust in the cultural characteristics of the host country will effectively help Chinese investors overcome the difficulty caused by cultural differences between the two countries, make it easier for them to be accepted by the host country, speed up the integration and even achieve better market performance in the future. Thus, Chinese OFDI could be largely promoted.

## Conclusion

In responding to the Chinese government’s call to “Going Global Strategy,” enterprises need to ensure that their cross-cultural investment can also bring benefits. Therefore, the cultural factors affecting Chinese OFDI are worth examining. The cultural distance between countries is a relative quality, and it is not easy to directly reflect how the host country’s culture affects Chinese OFDI decision-making. This paper focuses on the specific host country cultural characteristics, including cultural tolerance and trust specifically, and investigates their influence on Chinese OFDI. It is found that cultural tolerance and trust in the host countries are significantly positively correlated with Chinese OFDI. Therefore, Chinese enterprises should choose to enter countries with high cultural tolerance and trust for foreign direct investment to ensure safety and profitability. Further analysis reveals that the rule of law levels and economic freedom in the formal institution will affect the relationship differently. Using cultural distance to represent informal institutional distance, it is found that high cultural tolerance and trust promote Chinese OFDI in countries with higher cultural distance.

The complicated international environment, political, economic, and cultural factors are intertwined, bringing great uncertainty to Chinese OFDI. Because of the cultural difference between China and the host countries, it is necessary to seek common ground and shelve any differences. Therefore, to ensure higher investment returns with lower investment costs and take advantage of the more tolerant and trusting host country investment environments, these cultural characteristics’ influence on Chinese OFDI needs to be examined. At the same time, enterprises need to comprehensively analyze the host country’s formal and informal institutional environment to strengthen the effectiveness of Chinese OFDI policy formulation.

## Data Availability Statement

The datasets presented in this study can be found in online repositories. The names of the repository/repositories and accession number(s) can be found below: World Values Survey (WVS) website, Geert Hofstede website, World Bank database, CEPII database, and IMF database.

## Author Contributions

HL contributed to conception and design of the study. YW organized the database and performed the statistical analysis. YW and QZ wrote the first draft of the manuscript. JJ supervised the research and wrote sections of the manuscript. All authors contributed to manuscript revision, read, and approved the submitted version.

## Funding

This research was supported by the National Social Science Foundation of China (grant number: 19BJY100) and the Science and Technology Department of Sichuan Province (grant number: 2021JDR0075).

## Conflict of Interest

The authors declare that the research was conducted in the absence of any commercial or financial relationships that could be construed as a potential conflict of interest.

## Publisher’s Note

All claims expressed in this article are solely those of the authors and do not necessarily represent those of their affiliated organizations, or those of the publisher, the editors and the reviewers. Any product that may be evaluated in this article, or claim that may be made by its manufacturer, is not guaranteed or endorsed by the publisher.

## Footnotes

^1^The “Going Global Strategy” was formally proposed in 2000. It refers to a modern strong country strategy that Chinese enterprises make full use of “two markets, two resources” at home and abroad. Through OFDI, outward foreign project contracting, outward foreign labor cooperation, and other forms of active participation in international competition and cooperation, it aims to achieve the goal of sustainable development of China’s economy.

^2^The fieldwork for WVS 6 was conducted from 2010 to 2014, and the fieldwork for WVS 7 was conducted from 2017 to 2020. The WVS did not conduct fieldwork in 2015 and 2016. The data from 2015 and 2016 were the average of the survey results of WVS 6 (2010–2014) and WVS 7 (2017–2020).
